# Liposome armed with herpes virus-derived gH625 peptide to overcome doxorubicin resistance in lung adenocarcinoma cell lines

**DOI:** 10.18632/oncotarget.6013

**Published:** 2015-11-06

**Authors:** Emiliana Perillo, Stefania Porto, Annarita Falanga, Silvia Zappavigna, Paola Stiuso, Virginia Tirino, Vincenzo Desiderio, Gianpaolo Papaccio, Massimiliano Galdiero, Antonio Giordano, Stefania Galdiero, Michele Caraglia

**Affiliations:** ^1^ Department of Pharmacy and DFM Scarl - University of Naples “Federico II”, Naples, Italy; ^2^ Department of Biochemistry, Biophysics and General Pathology, Second University of Naples, Naples, Italy; ^3^ Department of Experimental Medicine, Section of Biotechnology and Medical Histology and Embryology, Second University of Naples, Naples, Italy; ^4^ Department of Experimental Medicine - Second University of Naples, Naples, Italy; ^5^ Sbarro Institute for Cancer Research and Molecular Medicine, Center for Biotechnology, College of Science and Technology, Temple University, Philadelphia, PA, USA; ^6^ Department of Medicine, Surgery and Neuroscience, University of Siena, Siena, Italy

**Keywords:** doxorubicin, drug resistance, membranotropic peptide, liposome, lung adenocarcinoma

## Abstract

New delivery systems including liposomes have been developed to circumvent drug resistance. To enhance the antitumor efficacy of liposomes encapsulating anti-cancer agents, we used liposomes externally conjugated to the 20 residue peptide gH625. Physicochemical characterization of the liposome system showed a size of 140 nm with uniform distribution and high doxorubicin encapsulation efficiency. We evaluated the effects of increasing concentrations of liposomes encapsulating Doxo (LipoDoxo), liposomes encapsulating Doxo conjugated to gH625 (LipoDoxo-gH625), empty liposomes (Lipo) or free Doxo on growth inhibition of either wild type (A549) or doxorubicin-resistant (A549 Dx) human lung adenocarcinoma. After 72 h, we found that the growth inhibition induced by LipoDoxo-gH625 was higher than that caused by LipoDoxo with an IC_50_ of 1 and 0.3 μM in A549 and A549 Dx cells, respectively. The data on cell growth inhibition were paralleled by an higher oxidative stress and an increased uptake of Doxo induced by LipoDoxo-gH625 compared to LipoDoxo, above all in A549 Dx cells. Cytometric analysis showed that the antiproliferative effects of each drug treatment were mainly due to the induction of apoptosis. In conclusion, liposomes armed with gH625 are able to overcome doxorubicin resistance in lung adenocarcinoma cell lines.

## INTRODUCTION

One of the most important goals in cancer treatment is the achievement of pharmacologically active concentrations of chemotherapy drugs in cancer tissues, avoiding drug distribution in healthy tissues. In fact, up-to-date a plethora of pharmacological weapons are available in order to control cancer growth but for none of these selectivity toward cancer cells [1] was demonstrated. An additional challenge is represented by the intracellular targeting of key molecules involved in cancer cell regulation once the efficacious delivery of drugs in cancer cells is achieved. The encapsulation of drugs in nanometric scaled biocompatible materials is a potential strategy for the accumulation of the drugs in the inflamed or tumor tissues through the use of the so-called and well-known enhanced retention and permeation effect (EPR) [1]. Once accumulated in tumor tissues nanocarriers can release the drug or can be internalized in cancer cells through endocytosis mechanism, resulting in intracellular trafficking in endosomes. Therefore, cleavage of the drugs out of nanocarriers and escape from the endosomes are further critical steps. A complication of cancer therapy is the potential development of chemoresistance that is due to the selection of cancer cell clones expressing molecules that protect tumour cells from anti-cancer agents [2, 3]. In this light, the overexpression of ATP binding cassette (ABC) transporters - such as *P*-glycoprotein (Pgp/ABCB1), multidrug resistance related proteins (MRPs/ABCCs) and breast cancer resistance protein (BCRP/ABCG2) - limits the intracellular retention and cytotoxicity of different chemotherapy drugs, conferring to tumor cells a multiple and cross-resistant phenotype known as multidrug resistance (MDR) [4]. Therefore, after the crossing of the fenestrated vessels, that are typical of cancer tissues, nanoparticles have also to overcome cell membrane barriers, release and retain the drug intracellularly at therapeutic levels for a desired time. Based on these, cancer cell membranes represent another critical barrier that affects both drug internalization and retention in cancer cells.

Among nanosystems used for drug delivery, liposomes have attracted great attention since they are ideal for loading and delivery of different molecules, therefore, offering novel opportunities for cancer treatment [5–7]. Benefits associated with liposomal drugs can derive from: i) protection of encapsulated drugs from chemical and biological degradation into the blood stream; ii) controlled release and reduced toxicity through decreased exposure of healthy tissues to anti-cancer drugs; iii) increased anti-tumor activity resulting from a relatively long systemic circulation time (especially in the case of PEGylated liposomes) [8–10]; iv) subsequent extended exposure and accumulation in growing tumor sites. To enhance the antitumor efficacy of liposomal drugs and to overcome the obstacle of the membrane barrier of cancer cells, many research groups are actively investigating how to improve liposome cell internalization through the addition of surface ligands. Recently, several cell penetrating peptides (CPPs) such as penetratin and Tat have been successfully used for the intracellular delivery of liposomes [5, 11, 12]. CPPs are a group of short, positively charged peptides with a potent ability to penetrate the membrane bilayer, which are well suited as drug delivery vehicles able to cross the biological barriers. The advantages of peptides as delivery enhancers include: i) their relatively small molecular weight, ii) easy synthesis, iii) relatively low cytotoxicity and immunogenicity, and iv) *in vivo* degradation [13]. Cationic cell-penetrating peptide-mediated endocytosis is one of the mechanisms by which drug carriers cross the membrane bilayer [14]; subsequently, the cargo is trapped in endosomes, eventually landing in lysosomes where its intracellular bioavailability is decreased. In order to avoid the endocytic pathway, it is of great importance to discover new molecules exploiting different mechanisms of uptake. Hydrophobic peptides that efficiently cross biological membranes, promoting lipid membrane-reorganizing processes represent a powerful alternative [15–17]. Viral-derived peptides can be useful as Trojan horses due to their intrinsic properties of inducing membrane perturbations [16–18].

The twenty residue peptide gH625, previously identified as a membrane-perturbing domain in the glycoprotein H (gH) of Herpes simplex virus 1 (HSV-1), is able to cross the membrane bilayer [19] and has been extensively used for vector-mediated strategies *in vitro*, which only partially involves the endocytic pathway [20–24]. We previously showed the drug carrier ability of gH625 functionalized DOPG based liposomes encapsulating Doxo and revealed differences between the uptake mechanisms of free and encapsulated Doxo [22]. Nuclear accumulation of free Doxo was attributed to drug diffusion, while encapsulated Doxo remained mostly in the cytoplasm with negligible nuclear accumulation [22].

Here, we investigated the *in vitro* anti-cancer activity of Doxo-encapsulating liposomes, constituted by soy phospholipids, cholesterol and 1,2-distearoyl-sn-glycero-3-phosphoethanolamine-N-[amino(polyethylene glycol)-2000] (DSPE-PEG), in order to improve biocompatibility and lead to a prolonged presence in the systemic circulation.

The anti-proliferative effects of liposomal formulations functionalized or not with gH625 were investigated on non-small cell lung cancer (NSCLC) A549 cells either sensitive or resistant to Doxo. The differential accumulation and the oxidative stress caused by the two different formulations in resistant and parental A549 cells were also evaluated.

## RESULTS

### Peptide synthesis and conjugation of gH625 to liposomes surface

The peptide gH625-Pra and the liposome component (C18)_2_L-N_3_ were synthesized according to standard solid phase peptide synthesis (SPPS) protocols with Fmoc/tBu (tBu = tert-butyl) chemistry. The alkyne moiety of gH625-Pra was introduced in the peptide sequence at the C-terminal position as L-propargylglycine. (C_18_)_2_L-N_3_ was synthesized on solid phase following a modified protocol of the classical Fmoc/tBu strategy [22]. Both gH625-Pra and (C_18_)_2_L-N_3_ were collected in good yields (∼ 40% and 85%, respectively) after HPLC-RP purification, and analyzed by mass spectrometry, ^1^H and ^13^C NMR spectroscopy (for (C_18_)_2_L-N_3_), and HPLC to confirm the compound identity and the purity.

**Figure 1:**
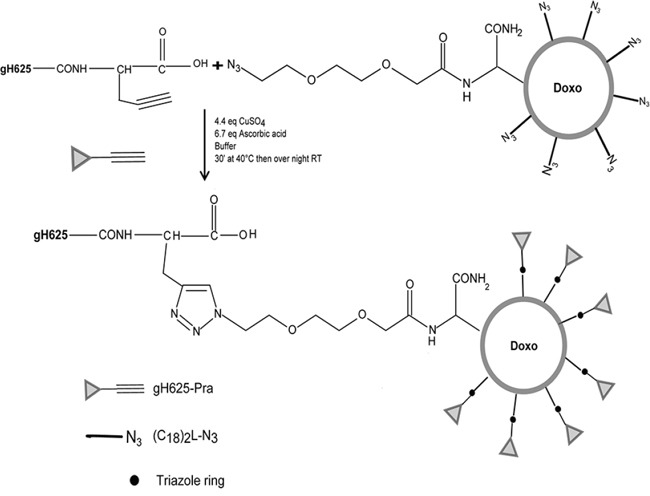
Scheme of the functionalization reaction of gH625 to LipoDoxo

The coupling of gH625 on the surface of preformed liposomes was performed by click chemistry (Figure 1). This procedure involves a copper^(I)-^catalyzed Huisgen 1,3-dipolar cycloaddition reaction of azides and alkynes yielding 1,4-disubstituted 1,2,3-triazole-linked conjugates [25]. The click reaction was performed in an aqueous solution and was catalyzed by Cu^I^ generated, *in situ*, by reduction of CuSO_4_ with ascorbic acid [26]. An equimolar mixture of NH_2_-gH625-Pra and azido functions on the liposome surface were used and the expected gH625-functionalized liposomes were obtained with a yield higher than 90% after 12 h at room temperature. In the absence of the copper catalyst no reaction was observed.

### Drug loading

Doxo was loaded into soy phospholipid mixture/cholesterol/DSPE-PEG/(C_18_)_2_L-N_3_ liposomes using the well-assessed procedure based on the ammonium sulphate gradient [27]; in particular, a solution containing Doxo was incubated under stirring for 30 min at 60°C. Subsequently, unloaded Doxo was removed using a Sephadex G50 column pre-equilibrated with HEPES-NaCl buffer (5 mM-100 mM) at pH 7.4. The drug/lipid weight ratio chosen for the loading experiments was 0.1. The drug loading content (DLC) was above 90% of the total.

The drug loaded liposomes were then efficiently modified with the gH625-Pra peptide according to the click-chemistry procedure used in the case of empty liposomes.

### Characterization of liposomes

Dynamic light scattering (DLS) measurements were performed on liposomes alone and on gH625 functionalized liposomes. Table 1 shows that all liposome solutions present a monomodal distribution with a polydispersity index (PDI) < 0.2 indicating a narrow and homogenous size distribution, optimal not only for the more effective extravasation of liposomes, but also for their longer retention in tumor tissues. The analysis of the zeta-potential shows a change between Lipo and LipoDoxo compared to LipoDoxo-gH625, which indicates a change in the surface of the liposomes upon functionalization with the peptide.

**Table 1:** Zeta potential, size, expressed as z-average, as measured by DSL and polydispersivity index (PDI) Data are expressed as means ± standard deviation (SD) of three separate experiments for each of two batch formulations, with at least 13 measurements for each.

Liposomes	Average Size (nm)	PDI	Zeta potential (mV)
***Lipo***	104.95 ± 1.63	0.17 ± 0.01	−7.40 ± 1.50
***LipoDoxo***	129.85 ± 1.82	0.15 ± 0.03	−7.43 ± 1.84
***LipoDoxo-gH625***	143.90 ± 0.64	0.14 ± 0.02	9.43 ± 1.61

### Release of doxorubicin from liposomes

**Figure 2:**
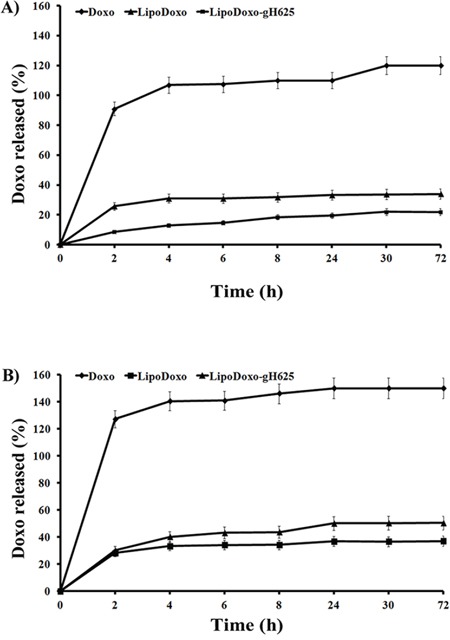
Release profile of doxorubicin from liposomes at 37°C in HEPES-NaCl buffer (a) and in HEPES-NaCl buffer with 50% FBS (b) Statistical analysis: LipoDoxo vs Doxo *P* < 0.01; LipoDoxo-gH625 vs Doxo *P* < 0.01.

The release of Doxo was carried out in HEPES-NaCl buffer or HEPES-NaCl buffer with 50% FBS and the results are presented in Figure 2. Free Doxo was used as control and its release rate was nearly 100% in 2 h, which means that the release of Doxo from dialysis membrane to buffer solution is not a restricting factor and the release of Doxo from the liposomes is the only rate limiting step. There were no pronounced differences in Doxo release (Figure 2) from LipoDoxo and LipoDoxo-gH625 at each time point, indicating that the decoration of the surface of the liposomes with gH625 did not substantially change the release kinetics of liposomes. The Doxo release from liposomes decorated and not with gH625 is less than 30% within 72 h, indicating their good stability.

### Effects of liposomes encapsulating doxorubicin conjugated or not with gH625 viral peptide on A549 and A549 Dx cell proliferation

**Figure 3:**
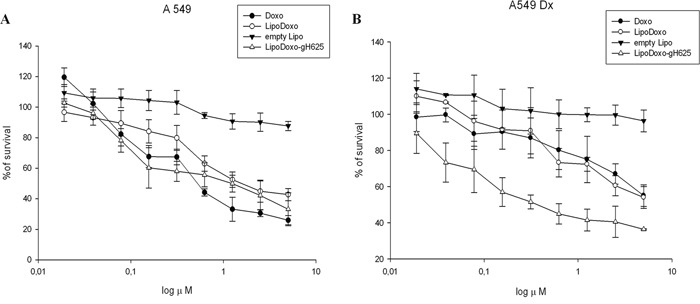
Evaluation of cell growth in lung adenocarcinoma cell line sensitive (A549) and resistant (A549 Dx) to doxorubicin after 72 h of treatment with Lipo, LipoDoxo, LipoDoxo-gH625 and doxorubicin (DOXO) (A–B) The figure shows representative experiments performed in triplicate with SDs. Statistical analysis: LipoDoxo vs LipoDoxo-gH625 *P* < 0.01; LipoDoxo vs Doxo *P* < 0.01; LipoDoxo-gH625 vs Doxo *P* < 0.01.

The effects of Doxo, empty liposomes (Lipo) and liposomes encapsulating Doxo conjugated or not with gH625 on the proliferation of either parental A549 or Doxo-resistant cells (A549 Dx) were evaluated by MTT assay as reported in “Materials and Methods”. Doxo, LipoDoxo and LipoDoxo-gH625 induced a dose-dependent growth inhibition in both cell lines after 72 h (Figure 3), while treatment with Lipo produced no significant cytotoxic effects in both cell lines (Figure 3). In Table 2, results are reported as concentrations inhibiting 50% of cell growth (IC_50_) after 72 h of treatment. The IC_50_ was reached with 0.8 μM and 5 μM of Doxo (Figure 3A and 3B, Table 2), with 1.6 μM and about 5 μM of LipoDoxo (Figure 3A and 3B, Table 2), with 1 μM and 0.3 μM of LipoDoxo-gH625 (Figure 3A and 3B, Table 2) in A549 and A549 Dx cells, respectively. These data suggested that A549 cells were more sensitive to the treatment with Doxo compared to A549 Dx, confirming the drug-resistant phenotype of this cell line. Both cell lines were more responsive to LipoDoxo-gH625 compared to LipoDoxo. In particular, LipoDoxo-gH625 was able to overcome resistance in A549 Dx compared to free Doxo. These data suggested that the conjugation of liposomes with gH625 probably facilitated the entry and retention of doxorubicin in both sensitive and drug-resistant tumor cell lines allowing an increase of cell growth inhibition.

**Table 2:** IC_50_ values of the different formulations Concentration inhibiting 50% of cell growth (IC_50_) reached after 72 h of treatment with Lipo, LipoDoxo, LipoDoxo-gH625, and Doxo in A549 and A549 Dx cells. Data are shown as mean ± SD

Compounds	IC50 ± SD A549	IC50 ± SD A549 DX
***Lipo***	> 5 μM ± 0.02	> 5 μM ± 0.01
***LipoDoxo***	1.6 μM ± 0.01	> 5 μM ± 0.02
***LipoDoxo-gH625***	1 μM ± 0.01	0.3 μM ± 0.04
***Doxo***	0.8 μM ± 0.03	> 5 μM ± 0.01

### Doxorubicin accumulation in A549 and A549 Dx cell lines

**Figure 4:**
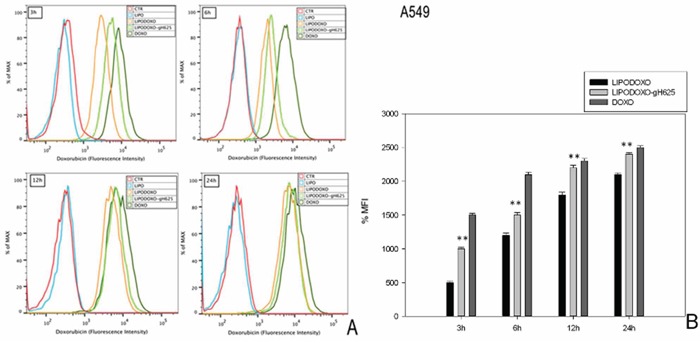
Doxorubicin accumulation in A549 cells after 3, 6, 12 and 24 h of treatment with LipoDoxo, LipoDoxo-gH625 and Doxo **A.** Flow cytometry overlay of Doxo fluorescence intensity. **B.** Histogram of Doxo mean fluorescence intensity (% of control). The bars represent means ± SD of three independent experiments. Asterisks indicate significant difference between LipoDoxo vs LipoDoxo-gH625 (**P < 0.01) (*P < 0.05).

**Figure 5:**
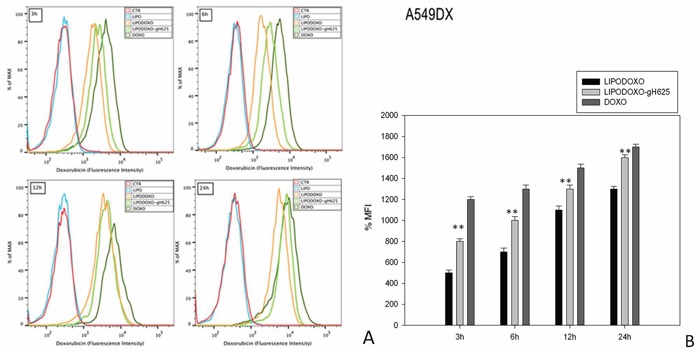
Doxorubicin accumulation in A549 Dx cells after 3, 6, 12 and 24 h of treatment with LipoDoxo, LipoDoxo-gH625 and Doxo **A.** Flow cytometry overlay of Doxo fluorescence intensity. **B.** Histogram of Doxo mean fluorescence intensity (% of control). The bars represent means ± SD of three independent experiments. Asterisks indicate significant difference between LipoDoxo vs LipoDoxo-gH625 (**P < 0.01) (*P < 0.05).

The accumulation of doxorubicin, free or encapsulated in liposomes conjugated or not with gH625, was investigated by flow cytometry analysis as reported in “Materials and Methods”. A time-dependent accumulation of free and encapsulated Doxo was observed in A549 and A549 Dx cells and the maximal levels were reached after 24 h (Figure 4 and 5). Moreover, LipoDoxo-gH625 induced in both cell lines a greater doxorubicin accumulation than LipoDoxo (Figure 4 and 5). In details, A549 Dx cells showed an early accumulation of doxorubicin after 3 h of treatment with both liposomal formulations and the accumulation was higher if compared to the one observed in parental A549 cells (Figure 4 and 5). This effect was more evident in resistant cells treated with LipoDoxo-gH625 that induced an increase of about 86.9% of MFI against an increase of about 64.3% of MFI induced in parental cells (Figure 5 and 4, respectively). Moreover, after 24 h we observed a two-fold increase of percentage of MFI in A549 Dx cells treated with LipoDoxo-gH625 if compared to those exposed to LipoDoxo (Figure 5). Similar data were also obtained in parental cells but to a lesser extent (Figure 4). On the other hand, free Doxo accumulation was more rapid and slightly higher than LipoDoxo-gH625 after 12 and 24 h. Therefore, these data suggested that the conjugation of liposomes with the viral peptide increased the retention of doxorubicin into the cells supporting the data obtained on the growth inhibition.

### Evaluation of oxidative stress in A549 and A549 Dx cells

**Figure 6:**
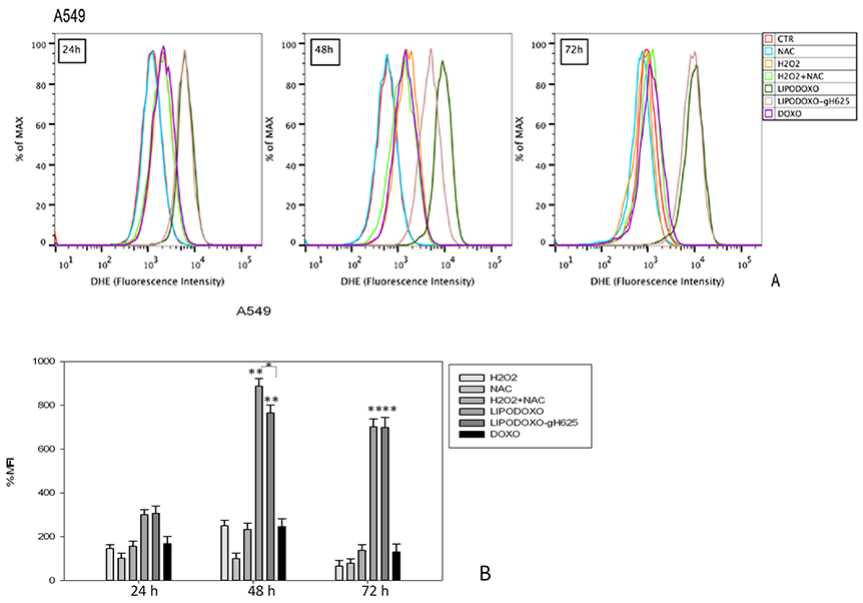
Evaluation of oxidative stress in A549 cells after 24, 48 and 72 h of treatment with LipoDoxo, LipoDoxo-gH625 and Doxo **A.** Flow cytometry overlay of dihydroethidium (DHE) fluorescence intensity. **B.** Histogram of DHE mean fluorescence intensity (% of control). The bars represent means ± SD of three independent experiments. Asterisks indicate significant difference between LipoDoxo vs LipoDoxo-gH625, LipoDoxo vs Doxo and LipoDoxo-gH625 vs Doxo (**P < 0.01) (*P < 0.05).

**Figure 7:**
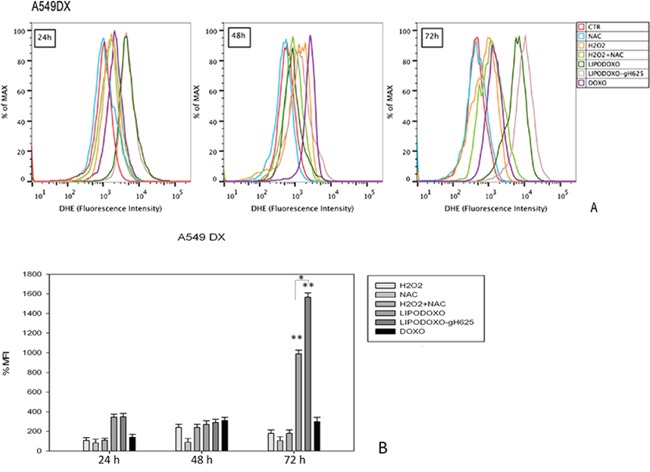
Evaluation of oxidative stress in A549 Dx cells after 24, 48 and 72 h of treatment with LipoDoxo, LipoDoxo-gH625 and Doxo **A.** Flow cytometry overlay of dihydroethidium (DHE) fluorescence intensity. **B.** Histogram of DHE mean fluorescence intensity (% of control). The bars represent means ± SD of three independent experiments. Asterisks indicate significant difference between LipoDoxo vs LipoDoxo-gH625, LipoDoxo vs Doxo and LipoDoxo-gH625 vs Doxo (**P < 0.01) (*P < 0.05).

The effects of Doxo and liposomes encapsulating doxorubicin conjugated or not with gH625 were also evaluated for analyzing the accumulation of superoxide anions (O_2_^−^) in either parental or Doxo A549 cells, as reported in “Materials and Methods”. In both cell lines free Doxo induced a time-dependent accumulation of superoxide anions (Figure 6 and 7) significantly lower compared to that induced by both liposomal formulations. In details, after 24 h LipoDoxo-gH625 and LipoDoxo induced a similar accumulation of O_2_^−^ and this effect was maintained up to the end of treatment (72 h) with LipoDoxo-gH625 differently from LipoDoxo on A549 cells (Figures 6). On the other hand, both formulations induced similar effects on A549 Dx cells after 24 h of treatment, but a significant increase of oxidative stress was observed after 72 h of treatment with LipoDoxo-gH625 if compared to the one induced by LipoDoxo (Figure 7). In both cell lines NAC had no effect on the increase of O_2_^−^ levels in contrast to H_2_O_2_ and acted as a scavenger in combination with H_2_O_2_ (Figures 6 and 7) decreasing the accumulation of superoxide anions. The data obtained on oxidative stress suggested a greater internalization of LipoDoxo-gH625 than LipoDoxo in A549 Dx after 72 h of treatment.

### Evaluation of cell death in A549 and A549 Dx cells

**Figure 8:**
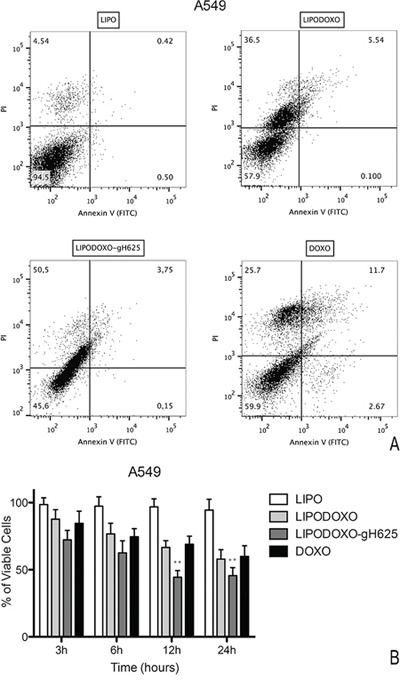
Evaluation of apoptosis in A549 cells by Annexin V/PI assay (flow cytometry) after 24 h of treatment with LipoDoxo, LipoDoxo-gH625 and Doxo **A.** Flow cytometry dot plots. **B.** Histogram of data expressed as percentage of viable cells, early/late apoptotic cells and necrotic cells after 3, 6, 12 and 24 h. The bars represent means ± SD of three independent experiments. Asterisks indicate significant difference between LipoDoxo-gH625 vs Doxo (**P < 0.01).

**Figure 9:**
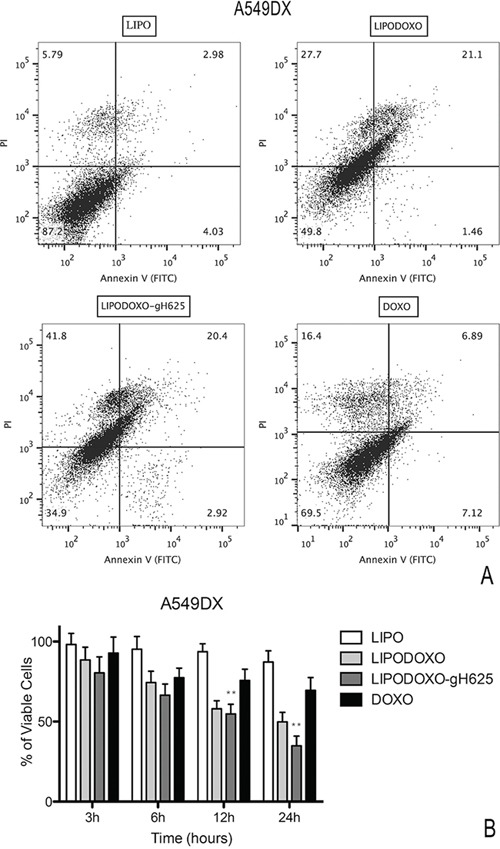
Evaluation of apoptosis in A549 Dx cells by Annexin V/PI assay (flow cytometry) after 24 h of treatment with LipoDoxo, LipoDoxo-gH625 and Doxo **A.** Flow cytometry dot plots. **B.** Histogram of data expressed as percentage of viable cells, early/late apoptotic cells and necrotic cells 3, 6, 12 and 24 h. The bars represent means ± SD of three independent experiments. Asterisks indicate significant difference between LipoDoxo-gH625 vs Doxo (**P < 0.01).

A further experiment was performed to evaluate the effects of Lipo, LipoDoxo-gH625, LipoDoxo and Doxo in inducing apoptosis or necrosis as reported in “Materials and Methods”. In agreement with the data obtained from the MTT assay, empty liposomes did not induce any significant toxic effects on both cell lines at any time-point tested (Figure 8 and 9). In contrast, LipoDoxo induced late apoptosis in about 5.5% of A549 cells while necrosis was recorded in about 36.5% of A549 cells after 24 h of treatment (Figure 8). This effect was potentiated also in doxorubicin-resistant cells by LipoDoxo-gH625 that caused about 3.8% of late apoptosis and about 50.5% of necrosis (Figure 8) On the other hand, LipoDoxo caused an accumulation of about 27.7% necrotic cells in resistant A549 Dx and about 21.1% of late apoptotic cells were recorded after 24 h. This effect was more marked when using LipoDoxo-gH625 with about 41.8% of necrotic cells and about 23.3% of apoptotic cells (Figure 9). Free doxorubicin induced more significant effects on A549 cells than on A549 Dx cells but to a lesser extent. In fact, it caused an accumulation of about 25.7% and 16.4% of necrotic cells in A549 and A549 Dx cells, respectively (Figure 8 and 9, respectively).

On the basis of these results, it can be suggested that LipoDoxo-gH625 induced more significant effects on cell death in both cell lines, but with different mechanisms. In fact, we have found that the main mechanism by which LipoDoxo caused cell death in parental A549 was necrosis while it caused apoptosis in doxorubicin-resistant counterpart. These effects were potentiated by LipoDoxo-gH625 in both experimental cell models (Figure 8 and 9).

### Intracellular distribution of Doxo in A549 and A549 Dx cells

**Figure 10:**
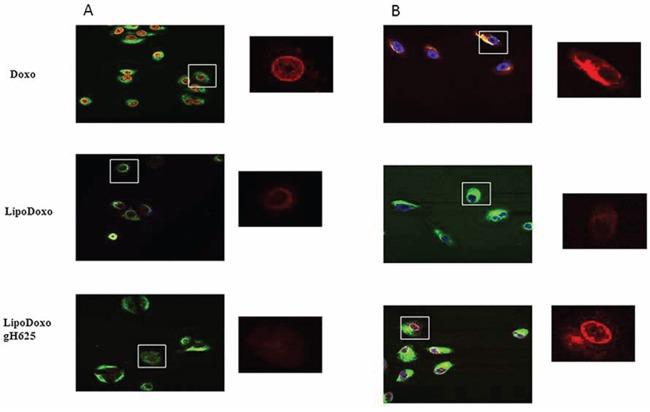
Confocal microscopy images of A549 (A) and A549 Dx (B) cells after 6 h incubation with free Doxo, LipoDoxo, or LipoDoxo-gH625 On the left, merged image (green, vimentin; red, doxo; blue, dapi). On the right, Doxo distribution.

**Figure 11:**
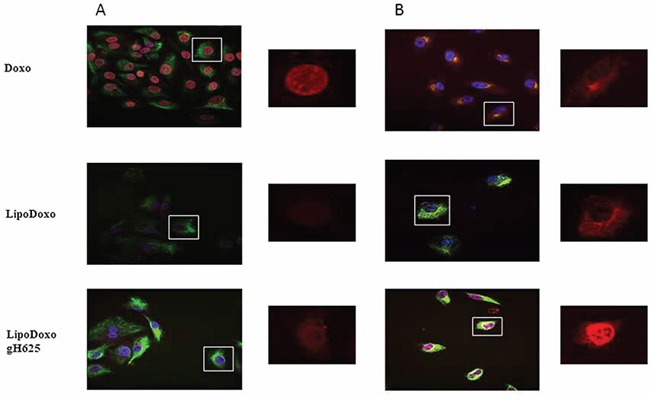
Confocal microscopy images of A549 (A) and A549 Dx (B) cells after 24 h incubation with free Doxo, LipoDoxo, or LipoDoxo-gH625 On the left, merged image (green, vimentin; red, doxo; blue, dapi). On the right, Doxo distribution.

In order to investigate the intracellular distribution of the different formulations, the Doxo fluorescence associated to the cells was evaluated by confocal laser scanning microscopy (CLSM). A549 and A549 Dx were incubated with IC50s of free Doxo, LipoDoxo and LipoDoxo-gH625 for 6 and 24 h. After 6 h, free Doxo and LipoDoxo-gH625 entered A549 cells and translocated into the nucleus as indicated by the red fluorescence in the center of the cell body (Figure 10A) while LipoDoxo accumulate in the cytoplasm, without entering into the nucleus (Figure 10A). In fact, cell nuclei are dark and only few red fluorescent spots, distributed in the cytoplasm, are visible. No fluorescence was observed in A549 treated with empty liposome as expected. On the other hand, in A549 Dx free Doxo was not able to enter into the nucleus accumulating in perinuclear region while CLSM results showed a widespread and intense fluorescence, with intranuclear red spots for cells incubated with LipoDoxo-gH625 (Figure 10B). Cells treated with LipoDoxo evidenced significant lower fluorescence intensity, with visible red spots into the cytoplasm (Figure 10B). We observed a time-dependent uptake of free and encapsulated Doxo and the maximal levels were reached after 24 h in both cell lines (Figure 11A and 11B) Therefore, the intracellular uptake of liposomes armed with gH625 could contribute to overcome resistance in lung adenocarcinoma cell lines.

## DISCUSSION

A major challenge in cancer therapy is the inability to deliver the chemotherapeutic agents to target tumor cells and tissues selectively, resulting in severe side effects to normal tissues and organs. Nanotechnology has the potential to promote delivery of drugs improving specificity and enhancing the uptake [28–30]. Moreover, the multidrug resistance (MDR) represents the principal mechanism by which many cancers develop resistance to chemotherapy, and poses the major obstacle to the successful clinical treatments of cancers [31, 32].

Due to these problems, it is highly important to explore alternative strategies for utilizing currently available drugs against MDR cancer cells. For many platforms designed for intracellular delivery, uptake occurs by an endocytic pathway. Although endocytic pathways can be very efficient in internalization, drug release and endosomal escape are key challenges in achieving high therapeutic efficacy.

Thus, current challenges include developing platforms with improved biodistribution, pharmacokinetic properties, and active targeting. While various active targeting strategies have been explored, there are no FDA-approved platforms [4, 33] highlighting the difficulties in reliably improving accumulation at the tumor site with active targeting.

Doxil is a liposomal formulation of doxorubicin, which was FDA-approved for AIDS-related Kaposi's sarcoma in 1995, for ovarian cancer in 1999, and for multiple myeloma in 2007. In 2013 the use of the generic version Lipodox was approved for treatment of ovarian cancer and Kaposi's sarcoma [1]. Doxil is formulated from a combination of fully hydrogenated soy phosphatidylcholine (HSPC), cholesterol, and a lipid with a polyethylene glycol (PEG) head group (DSPE-PEG2k) in a mole ratio of 56.4:38.3:5.3. The DSPE-PEG provides a polymer coating that can inhibit protein adhesion and prolong evasion of mononuclear phagocytic system (MPS) [4, 12]. Such coatings lead to long circulation half-times (3–4 days in humans) and are essential to achieve significant passive accumulation at a tumor site.

Successful design of nanosystems to treat tumor effectively requires the appropriate surface modification to enhance the uptake; in fact, the activity of cytotoxic drugs that are internalized by cells depends also on their concentration and availability in the cell cytosol and nuclei. Peptide gH625 represents a novel opportunity to overcome the known limits of classical CPPs and proved to be successful for delivery of several molecules, therefore was chosen in our study to increase the cell internalization of liposomes into cells.

The different phospholipids used to prepare the liposomes investigated in the present study are of paramount importance to determine the uptake and mechanism of internalization. We have previously reported preliminary results on the internalization of DOPG liposomes decorated on their surface with gH625 and we proved that gH625 allowed to modify the mechanism of internalization of Doxo, but the eukaryotic membranes are zwitterionic; thus DOPG, being negatively charged, does not well mimics eukaryotic cell membranes. In this study we used liposomes with enhanced biomimetics characteristics (phospholipids derived by soy) and functionalized on their external surface with PEG.

Moreover, we examined the potential of gH625 targeted liposomes to efficiently deliver encapsulated Doxo into target cells and, consequently, to determine significant cytotoxicity in drug-sensitive cells (A549) as well as in drug-resistant cells (A549 Dx). The results described in the present manuscript, suggest that LipoDoxo-gH625 can be used as an *in vivo* drug delivery system to bypass *P*-gp-mediated efflux of Doxo from the cells, thus circumventing drug-resistance of tumor cells. The crucial finding of this study was that only the LipoDoxo-gH625, but not LipoDoxo or free Doxo, led to substantially enhanced uptake and nuclear accumulation of encapsulated Doxo in both A549 and A549 Dx cells but at a higher extent in Doxo-resistant cells. The results described suggest that LipoDoxo-gH625 is internalized by cells more efficiently than LipoDoxo. This was supported by our confocal microscopic study showing that the LipoDoxo-gH625 was extensively accumulating within the cell and, above all in the nucleus. This clearly indicates that gH625 affects the process of liposome internalization via receptor-mediated endocytosis. The presence of gH625 on the surface of liposomes favored their uptake in both sensitive and drug-resistant tumor cell lines allowing an increase of cell growth inhibition: in fact, a greater quantity of Doxo from functionalized liposomes is accumulated into cells compared to not functionalized ones; however, even with the gH625-targeted liposomes the extents of Doxo accumulation were slightly lower than those of free Doxo as shown by FACS analysis. On the other hand, we observed similar cytotoxicities of free Doxo alone and gH625 targeted-liposomes on A549 cells but LipoDoxo-gH625 was much more potent on A549 Dx growth inhibition. Moreover, LipoDoxo-gH625 was able to induce increased cell death and oxidative stress compared to LipoDoxo and free Doxo on both cell lines. In particular, LipoDoxo and LipoDoxo gh625 induced necrosis in parental cells and apoptosis in resistant cells. These liposomal formulations are able to induce apoptosis in resistant cells that are known to be less prone to undergo apoptosis induced by conventional drugs.

As an inhibitor of mitochondrial complex-I, Doxo [34] produces ROS that can result in oxidative stress and subsequent apoptosis. Opening of the mitochondrial permeability transition pore (MPT) and the disruption of mitochondrial transmembrane potential are also central steps in the apoptotic cell death signaling pathway. However, it has recently become evident that mitochondria can also play a critical role in primary necrosis. Damage to mitochondria may result in disruption to the mitochondrial electron transport chain, which would, in turn, result in reduced ATP production and consequently in a disruption of the bioenergetic state of the cells leading to necrotic cell death [35]. The mechanisms underlying the cell death mode switch have been analyzed in several cell types, and a few conclusions have been proposed, including decrease in the intracellular level of ATP [36]. These liposomal formulations by generating high levels of ROS that probably reduced ATP pools induced a switch between apoptosis and necrosis.

At present, it is not fully understood how ROS trigger both necrosis and apoptosis, but the ROS quantity seems to be critical for determining the cell death mode. A higher oxidative stress is obtained after treatment with functionalized liposomes for 72 h indicating that probably also other mechanisms contribute to the activity in presence of gH625. In addition, drug-free liposomes displayed no cytotoxicity against A549 and A549 Dx at the evaluated concentrations, indicating that these formulations were unable to induce growth inhibition or cell death.

This may mean that the amount of Doxo delivered in the cells is less important in determining the cytotoxic potential of the drug than the mechanism by which it is delivered. We tentatively propose that Doxo entering the cell by means of Lipodoxo-gH625 is delivered deeper into the cell interior than free Doxo, conceivably closer to the nucleus. As a consequence, a major fraction of the drug escapes expulsion by the *P*-gp pump and is sequestered by the nearby nucleus, where it can exert its cytostatic action. Riganti group characterized A549 and A549 Dx with regard to the ABC transporter expression [37]. In some instances, however, the expression of these drug efflux pumps has no effect on the total cellular accumulation of multidrug resistance substrates. The net intracellular accumulation of the anthracycline doxorubicin, for example, is unaltered by the expression of either MRP1 or Pgp, even if the cellular toxicity of the drug is mitigated by the presence of these transport proteins [38]. Rajagopal *et al*. [38] showed that the expression of MRP1-EGFP resulted in decreased nuclear accumulation of doxorubicin. In particular, CLSM showed that LipoDoxo-gH625 efficiently accumulated in A549 Dx nucleus compared to LipoDoxo after 6 h and at higher extent after 24 h while free Doxo was not able to enter the nucleus and accumulates in the perinuclear region. FACS analysis demonstrated that intracellular accumulation of free Doxo in A549 Dx cells was slightly higher than LipoDoxo-gH625 accumulation analysis but only LipoDoxo-gH625 was able to reach cell nucleus inducing apoptosis and oxidative stress.

Nevertheless, we cannot exclude that at least some drug release occurs already during the binding stage at the cell surface. Thus, the physicochemical properties of the liposomes, in addition to the presence of the targeting device, may be a key factor in determining the extent of intra-nuclear Doxo accumulation, and hence the cytotoxic efficiency of the drug.

Above all in the MDR cells the combination of a high rate of internalization and the subsequent release of Doxo might overload the capacity of drug efflux by *P*-gp, thus contributing to the relief from drug resistance.

## CONCLUSION

In conclusion, Doxo encapsulated in functionalized liposomes accumulated in the nuclei of Doxo-resistant cancer cells indicating that the peptide gH625 was probably able to induce a greater and more rapid internalization also in resistant cells, which could contribute to circumvent multidrug resistance and improve the *in vivo* pharmacological advantages of long-circulating liposomal delivery systems [39]. Further details have been shed on the mechanism of internalization promoted by gH625 which clearly indicates that this translocating peptide hold promise for the development of a platform for cancer therapy.

## MATERIALS AND METHODS

Fmoc-protected amino acid derivatives, coupling reagents, and Rink amide *p*-methylbenzhydrylamine (MBHA) resin were purchased from Calbiochem-Novabiochem (Laufelfingen, Switzerland). Fmoc-l-propargylglycine (Fmoc-Pra-OH) was purchased from Polypeptide (Strasbourg, France). Doxorubicin hydrochloride and the other chemicals were purchased from Sigma–Aldrich, Fluka (Buchs, Switzerland), or LabScan (Stillorgan, Ireland) and were used as received, unless otherwise stated. All phospholipids were purchased from Avanti Polar Lipids (Alabaster, AL); in particular the soybean phospholipid mixture is composed of Soy PC (3.8 mg) Soy PE (3.0 mg) Soy PI (1.8 mg) Soy PA (0.7 mg) Soy LPC (0.7 mg) with 0.1% butylated hydroxytoluene (BHT). Culture medium DMEM, fetal bovine serum and tissue culture plastic ware were purchased from Microtech (Naples, Italy). Annexin V-FITC Apoptosis Detection Kit was purchased from eBioscience (San Diego, CA, USA). Dihydroethidium (DHE) was purchased from Sigma-Aldrich (Milan, Italy).

### Solid-phase synthesis of gH625 and Azide-AdOO-Lys(C(O)CH_2_CH_2_C(O)N-(C_18_H_37_)_2_)-amide

The peptide was synthesized using standard solid-phase 9-fluorenylmethoxycarbonyl (Fmoc) procedures with a Syro I MultiSynThec GmbH (Wullener, Germany) automatic synthesizer using a Rink amide MBHA resin (substitution: 0.51 mmol/g; synthesis scale: 20 μmol). The peptide was obtained by repeated cycles of deprotection and coupling. Coupling: 4 equiv of Fmoc-protected amino acids relative to resin loading, HBTU (0.5 M in DMF, 4 equiv), HOBt (0.5 M in DMF, 4 equiv), and DIPEA (2 M in DMF, 8 equiv). Deprotection: 30% piperidine (v/v) in DMF for 10 min (2 times). All couplings were performed twice for 0.5 h. Fmoc-Pra-OH was coupled once for 45 min with 2 equivalents of PyBop/HOBt and 4 equivalents of DIPEA. The peptide was fully deprotected and cleaved from the resin with a solution of TFA/water/anisole/thioanisole 93.5/2.5/2.0/2.0 at room temperature for 300 min, and then precipitated with ice-cold ethyl ether, filtered, dissolved in water, and lyophilized. The crude peptide was purified by RP-HPLC on a LC8 Shimadzu HPLC system (Shimadzu Corporation, Kyoto, Japan) equipped with a UV lambda-Max Model 481 detector using a Phenomenex (Torrance, CA) C_18_ (300 Å, 250 × 21.20 mm, 5 μ) column eluted with H_2_O/0.1% TFA (A) and CH_3_CN/0.1% TFA (B) from 20–80% over 20 min at a flow rate of 20 mL min^−1^. Purity and identity were assessed by analytical LC-MS analyses using Finnigan Surveyor MSQ single quadrupole electrospray ionization (Finnigan/Thermo Electron Corporation San Jose, CA), column: C_18_-Phenomenex eluted with H_2_O/0.1% TFA (A) and CH_3_CN/0.1% TFA (B) from 20–80% over 10 min at a flow rate of 0.8 mL min^−1^. The final yield of purified peptide was ∼40%. Azide-AdOO-Lys(C(O)CH_2_CH_2_C(O)N-(C_18_H_37_)_2_)-amide ((C_18_)_2_L-N_3_) monomer was synthesized on the solid phase under standard conditions using the Fmoc/tBu strategy as previously reported [22].

### Liposomes preparation

Liposomes were prepared by the thin lipid film hydration procedure. Mixed aggregates of soy phospholipid mixture/cholesterol/DSPE-PEG/(C_18_)_2_L-N_3_ (57:28:5:10 molar ratio) were prepared by dissolving the lipids in a small amount of chloroform, and subsequently evaporating the solvent by slowly rotating the tube containing the solution under a stream of nitrogen and lyophilized overnight. In this way a thin film of amphiphiles was obtained. The dry lipid film was suspended in HEPES-NaCl buffer (5 mM-100 mM) at pH 7.4 by vortexing for 1 h; then the lipid suspension was freeze–thawed ten times and extruded ten times thought a polycarbonate membrane with 100 nm pore size using a thermobarrel extruder (Northern Lipids).

### Functionalization of liposomes with gH625

This procedure of functionalization of liposomes involves a copper(I)-catalyzed Huisgen 1,3-dipolar cyclo-addition reaction of azides and alkynes yielding 1,4-disubstituted 1,2,3-triazole linked conjugates. The unreactive nature of both azides and alkynes towards any other functional group present in the biomolecules, as well as the thermal and hydrolytical stability of their cycloaddition product make this reaction particularly appealing for liposome functionalization with peptides. The click reaction was carried out on preformed soy phospholipid mixture/cholesterol/DSPE-PEG/(C_18_)_2_L-N_3_ liposomes adding CuSO_4_•5H_2_O (4.4 equiv), ascorbic acid (6.7 equiv), and the peptide derivative (1 equiv). In particular, solutions containing CuSO_4_•5H_2_O (60.5 mM, solution A), ascorbic acid (81.4 mM, solution B), and the alkyn-modified peptide (1 mM, solution C) were freshly prepared in water. The reaction was catalyzed by Cu(I) generated, *in situ*, by reduction of CuSO4 with ascorbic acid.

Solution A (11.6 μL), solution B (13.2 μL), and solution C (145.4 μL) were added to a suspension of azido-functionalized liposomes in HEPES buffer (400 μL). The concentration of solution C was determined measuring the absorbance with a UV/Vis Jasco V-5505 spectrophotometer.

The reaction mixture was stirred at 40°C for 30 min and left overnight at room temperature. After the conjugation step the liposomes were purified by exclusion chromatography on a 1 × 18 cm Sephadex G-50 (Amersham Biosciences) column pre-equilibrated with HEPES buffer.

### Doxo encapsulation in liposomes

Doxorubicin was remote-loaded in soy phospholipid mixture/cholesterol/DSPE-PEG/(C_18_)_2_L-N_3_ liposomes through the ammonium sulphate gradient method and the free Doxo was removed by gel filtration. Briefly, the liposomal film was suspended in an ammonium sulphate solution (250 mM) at pH 5.5. The external buffer was removed by ultracentrifugation at 40000 rpm (Beckman Optima L-70 Ultracentrifuge) at 4°C for 3 h, and liposomes were resuspended in HEPES-NaCl buffer (5 mM-100 mM) at pH 7.4. A Doxo solution in water was added to the liposomal solution. This suspension was stirred for 30 min at 60°C. The unloaded Doxo was removed using a Sephadex G50 column and the Doxo concentration was determined by UV spectroscopy measuring the absorbance at λ = 480 nm. The drug loading content (DLC, defined as the weight ratio of encapsulated Doxo vs. the amphiphilic moieties) was quantified by subtraction of the amount of Doxo removed from the total amount of Doxo loaded. Finally, Doxo pre-loaded liposomes were modified with gH625 using the click-chemistry reaction procedure, as reported above.

### Particle size and zeta potential analyses

The hydrodynamic diameters (D_H_) and polydispersity index (PDI) of liposomes (Lipo), Doxo-loaded liposomes (LipoDoxo) and Doxo-loaded liposomes-gH625 (LipoDoxo-gH625) were measured using dynamic light scattering (DLS) (Malvern Zetasizer Nano ZS, Malven, UK). The zeta potential of LipoDoxo-gH625 was determined using a Malvern NanoZ (Malvern Zetasizer Nano ZS, Malven, UK). The analysis were performed with He–Ne laser 4 mW operating at 633 nm at scattering angle fixed at 173° and at 25°C. The results were determined three times for each sample and each measurement was performed at least in triplicate.

### *In vitro* Doxo release from liposomes

The *in vitro* release of Doxo from LipoDoxo and LipoDoxo-gH625 was determined using a dialysis method. Briefly, free Doxo and Doxo-loaded liposomes (with free Doxo removed) were placed in a dialysis bag (MW cut off of 1000 Da) and dialyzed against HEPES-NaCl and HEPES-NaCl with 50% fetal bovine serum under continuous stirring at 37°C. At predetermined time intervals, aliquots were withdrawn and replaced with an equal volume of fresh medium. The Doxo concentrations were calculated based on the fluorescence absorbance intensity of Doxo excited at 485 nm using a previously established calibration curve. The cumulative amount of Doxo released over the 72 h was quantified, and results were plotted against time.

### Cell culture

Human lung adenocarcinoma cell line wild type (A549) and doxorubicin (Doxo)-resistant (A549 Dx) were kindly provided by Chiara Riganti, MD (Department of Genetics, Biology and Biochemistry, University of Turin, Italy). Both cell lines were grown in DMEM supplemented with 10% heat-inactivated fetal bovine serum, 20 mM HEPES, 100 U/mL penicillin, 100 mg/mL streptomycin, 1% L-glutamine and 1% sodium pyruvate. The resistance to Doxo in A549 Dx cell line was maintained by administering 10 nM of Doxo in alternating steps. A549 and A549 Dx cells were cultured at a constant temperature of 37°C in a humidified atmosphere of 5% carbon dioxide (CO_2_).

### Cell proliferation assay

The evaluation of cell proliferation was performed on human lung adenocarcinoma cell line wild type and doxorubicin resistant in the presence of increasing concentrations of LipoDoxo, LipoDoxo-gH625 and Lipo in a range of 5–0.04 μM or free Doxo in a range of 3–0.02 μM. A549 and A549 Dx cells were seeded in 96-well plates in a number of 25 × 10^2^ per well. The growth inhibition was assessed by MTT viability assay after 72 h of treatment as previously described [40]. Then the concentrations inhibiting 50% of cell growth (IC_50_) were obtained and these values were used for subsequent experiments. MTT assay was carried out by triplicate determination on at least three separate experiments. All data are expressed as mean ± SD.

### Flow cytometric analysis of Doxo accumulation

The accumulation of Doxo was analyzed by FACSAria™ (BD Bioscences) after treating A549 and A549 Dx cells with a fixed concentration (10 μM) of Lipo, LipoDoxo, LipoDoxo-gH625 and Doxo. Briefly, A549 and A549 Dx cells were seeded in 6-well plates in a number of 2 × 10^5^ cells per well and were treated 24 h later with each formulation and free Doxo. After 3, 6, 12 and 24 h of treatment cells were trypsinezed, washed twice with PBS 1X and pellets were resuspended in 500 μL of PBS 1X. Doxo fluorescence associated to the cells was measured using FL2 channel and calculated as mean fluorescence intensity (MFIs) for each sample. For each sample, 2 × 10^4^ events were acquired. Analysis was carried out by triplicate determination on at least three separate experiments.

### Flow cytometric analysis of oxidative stress

The evaluation of ROS accumulation was detected using dihydroethidium (DHE), a specific marker for the determination of reactive oxygen species, in detail superoxide anion. Once oxidized within the cell, DHE is converted into ethidium (HE) and emits at the wavelength of 605 nm. Briefly, A549 and A549 Dx cells were seeded in 6-well plates in a number of 2 × 10^5^ cells per well and were treated 24 h later with concentration inhibiting 50% of cell growth of each formulation and Doxo. A549 and A549 DX cells were also treated with 500 μM of H_2_O_2_, which is able to induce superoxide anion formation, 2000 μM of N-acetylcysteine (NAC) as antioxidant agent, and H_2_O_2_ in combination with NAC. At the end of treatments, A549 and A549 Dx cells were incubated for 1 h with 20 ng/mL DHE stock solutions (2.5 mg/mL). At the time of processing, cells were trypsinized, washed twice with PBS 1X and the pellet was resuspended in 500 μl of PBS 1X. The dye accumulation was measured using FL2 channel by BD FACSAria™ (BD Bioscences) analysis and calculated as mean fluorescence intensity (MFIs) for each sample. For each sample, 2 × 10^4^ events were acquired. Analysis was carried out by triplicate determination on at least three separate experiments.

### Flow cytometric analysis of apoptosis

Apoptotic cell death was analysed by Annexin-V–FITC staining and by propidium iodide (PI) detection systems (eBioscences, Vienna, Austria). Briefly, A549 and A549 Dx cells were seeded in 6-well plates in a number of 2 × 10^5^ cells per well and were treated 24 h later with concentration inhibiting 50% of cell growth of LipoDoxo, LipoDoxo-gH625, Doxo and 5 μM of Lipo (concentration proved to be not toxic). After 3 h-6 h-12 h-24 h of treatment cells were trypsinezed, washed twice with PBS 1X and pellets were resuspended in 200 μL Binding Buffer 1X. Then, 5 μL Annexin V-FITC were added to 195 μL cell suspension, mixed and incubated for 10 min at room temperature. Cells were washed with 200 μL Binding Buffer 1X, resuspended in 190 μL Binding buffer 1X and 10 μL Propidium Iodide (20 μg/mL) was added. The detection of viable cells, early apoptosis cells, late apoptosis cells and necrotic cells were performed by FACSAria™ (BD Bioscences) by using a blue laser (488 nm) with three detection filters 480/10 (SSC/FSC), 530/30 (FITC) and 610/20 (PI) [41]. For each sample, 2 × 10^4^ events were acquired. Analysis was carried out by triplicate determination on at least three separate experiments.

### Evaluation of intracellular distribution of Doxo by confocal microscopy

After 6 and 24 of incubation of A549 and A549 Dx cells with fluorescent Lipo, cells were fixed for 20 minutes with a 3% (w/v) paraformaldehyde (PFA) solution and permeabilized for 10 minutes with 0.1% (w/v) Triton X-100 in phosphate-buffered saline (PBS) at room temperature. To prevent nonspecific interactions of antibodies, cells were treated for 2 h in 5% bovine albumin serum (BSA) in PBS, then cells were incubated with a specific mouse monoclonal Ab raised against vimentin (1:1,000 in blocking solution, 3% (w/w) BSA in TBS-Tween 0.1%, Sigma) for 2 h at 37° C. After several washes, cells were incubated with a secondary IgG goat anti-mouse antibody (Alexa Fluor 488, Life Technologies, Carlsbad, CA) diluted 1:1,000 in blocking solution for 1 h at room temperature. The slides were mounted on microscope slides by Mowiol. The analyses were performed with a Zeiss LSM 510 microscope equipped with a plan-apochromat objective X 63 (NA 1.4) in oil immersion. The fluorescences of the Doxo and Alexa Fluor 488 were collected in multi-track mode using BP550–625 and LP650 as emission filters, respectively.

### Statistical analysis

All data are expressed as mean ± SD. Statistical analysis was performed by analysis of variance (ANOVA) with Neumann-Keul's multiple comparison test or Kolmogorov-Smirnov where appropriate.
